# Citywide park renovations and changes in perceived stress: a quasi-experimental study among low-income communities in New York City

**DOI:** 10.1186/s12889-025-23639-7

**Published:** 2025-07-19

**Authors:** Rachel L. Thompson, Katarzyna E. Wyka, Kelly R. Evenson, Lorna E. Thorpe, Glen D. Johnson, Brian T. Pavilonis, Terry T.-K. Huang

**Affiliations:** 1 Center for Systems and Community Design, Graduate School of Public Health and Health Policy, City University of New York (CUNY), 55 West 125th Street, New York, NY 10027 USA; 2 NYU-CUNY Prevention Research Center, New York, NY USA; 3 Department of Environmental, Occupational, and Geospatial Health Sciences, Graduate School of Public Health and Health Policy, City University of New York (CUNY), 55 West 125th Street, New York, NY 10027 USA; 4 Department of Epidemiology, Gillings School of Global Public Health, University of North Carolina–Chapel Hill, 135 Dauer Drive, Chapel Hill, NC 27599 USA; 5 Department of Population Health, Grossman School of Medicine, New York University (NYU), 550 1st Avenue, New York, NY 10016 USA

**Keywords:** Park renovation, Green space, Stress, Mental health, Health equity, Built environment

## Abstract

**Background:**

Quality parks have the potential to promote well-being and health equity in urban communities through reduced stress, yet high-quality epidemiological evidence is limited. This quasi-experimental study measured associations between park renovation and changes in perceived stress among low-income adults in New York City.

**Methods:**

Pre- and post-renovation data on the Perceived Stress Scale and park use from 162 adults living near (< 0.3 miles) 31 renovated parks and 151 adults living near 21 sociodemographically matched control parks were analyzed. Linear mixed-effects difference-in-difference (DID) regression measured the association between park renovation and change in perceived stress (post-pre) in the overall sample and stratified by baseline sociodemographics. Additional models explored the interaction of post-renovation park use frequency [high (≥ once/week), low (< once/week)] and intervention status on changes in perceived stress.

**Results:**

Overall, changes in perceived stress were similar between intervention and control groups [DID = 0.28 (95% CI -1.48, 2.03)]. However, park renovation was associated with a significant decrease in perceived stress among divorced/separated/widowed participants [DID = -4.22 (95% CI -7.92, -0.53)] and middle-aged participants (35-49y) with high park use [DID = -4.46 (95% CI -8.28, -0.64)]. Among intervention but not control participants, those with high park use experienced a significantly larger decrease in perceived stress compared to those with low park use [DID = -2.92 (95% CI -5.36, -0.47)].

**Conclusions:**

In one of the first and largest studies on park quality improvement and mental health, park renovation near one’s home was associated with decreased perceived stress among divorced/separated/widowed adults and middle-aged frequent park users. Frequent users of renovated parks experienced a larger drop in perceived stress than infrequent users, suggesting that high-quality parks may be an important pre-condition to the benefits of frequent park use on stress reduction.

**Supplementary Information:**

The online version contains supplementary material available at 10.1186/s12889-025-23639-7.

## Background

Stress in the United States (U.S.) has been characterized as a national mental health crisis [1]. In 2023, 24% of adults in the U.S. rated their average level of stress between 8 and 10 on a scale of 1 (little to no stress) to 10 (a great deal of stress) – up from 19% in 2019 [2]. The increased prevalence of high perceived stress levels in 2023 may be attributable to lingering post-traumatic effects of the global pandemic, as well as widespread collective trauma experienced by Americans due to economic challenges, political division, racial and ethnic discrimination, global conflict, and climate-related disasters [2].

The biological manifestation of acute stress – the “fight-or-flight” response – is an evolutionary survival mechanism that has historically helped humans survive threats from predators and other dangers. However, frequent or ongoing activation of this response by modern-day stressors can result in pathophysiological changes, such as high blood pressure and vascular hypertrophy [3], arterial plaque buildup [4], immune suppression [5], hormonal dysregulation [6] and alterations in brain structure [7]. Prolonged pathophysiological changes from chronic stress can contribute to the onset of various physical and mental health conditions, including cardiovascular disease [8], diabetes [9, 10], anxiety [11], depression [12] and premature mortality [13].

In New York City (NYC), mental health disorders are highly prevalent – with 20% of adults (over 1.3 million people) reporting a mental health disorder in 2018–2019 [14, 15]. In urban contexts such as NYC, stress and poor mental health are prominent health equity concerns. A survey of adult New Yorkers in 2023 found higher rates of severe mental health symptoms among Black, Latino/a, Middle Eastern or North African, and multiracial residents compared to White residents [14]. The prevalence of severe mental health symptoms was also found to be higher among young adults (18-24y), sexual and gender minorities, those with less than a Bachelor’s degree, and individuals living in high-poverty neighborhoods [14]. Inequalities, poverty, and systemic racism exacerbate health disparities within cities, often resulting in concentrated areas of deprivation where residents suffer from limited access to resources and higher rates of chronic diseases [16, 17]. Built environment factors related to historical disinvestment in minoritized communities, such as neglect in services and maintenance of buildings, streets, and parks, may also play an important role in generating and sustaining mental health disparities in urban areas [14].

Neighborhood parks are key features of the urban built environment that have the potential to provide respite from the stress of city life. The aesthetic and tranquil nature of these spaces offer potential mental health benefits, including stress reduction and enhanced mood, contributing to overall psychological well-being [18, 19]. Residential proximity to urban parks has been associated with lower psychological distress, with residents living withing walking distance of a park experiencing the greatest mental health benefits [20]. The benefits of urban green spaces may also include mitigation of negative mental health impacts during crisis situations such as the COVID-19 pandemic [21]. Various mechanisms have been proposed to explain how urban green spaces may influence mental health, including increased physical activity [22, 23], increased social interaction [24], strengthened neighborhood ties [25] and improved quality of life [26].

Park usage patterns may also play a moderating role in the association between urban greenspace exposure and mental health benefits, with evidence suggesting that a greater frequency or duration of park use is linked to better physical and mental health outcomes [27–29]. Despite a growing body of research on the topic, a definitive positive link between urban greenspace exposure and mental health benefits remains elusive, largely due to a reliance on weak observational study designs, inconsistent measures of greenspace exposure, diverse study populations, and other complex contextual factors that may not be adequately captured [30].

The quality of public parks and green spaces is also a critical factor when considering the relationship between parks and health. Parks that are perceived as high-quality, well-maintained, and safe tend to be used more frequently, allowing the potential health benefits of parks to take place [31, 32]. Parks in poor condition or perceived as dangerous places may have an opposite association with health outcomes and quality of life [25, 33]. Disparities in access to safe, quality parks tend to exist in the very communities that could benefit most from them, as studies have shown that neighborhood parks within low-income communities tend to be fewer in number, have less amenities, and be less well-maintained than parks in high-income communities [34–36].

Disparities in mental health and in access to quality green spaces pose intersecting health equity concerns that could potentially be addressed through innovative, large-scale built environment interventions. One such intervention is the Community Parks Initiative (CPI) - a major ongoing citywide policy initiative providing infrastructure improvements to dozens of parks situated within low-income, densely populated, and rapidly growing communities in NYC with a history of disinvestment [37]. To date, CPI has led to the complete redesign and renovation of over 60 neighborhood parks across NYC. Renovated parks have benefitted from several infrastructure improvements, including expanded green areas and shading, better lighting and aesthetics, upgraded walking paths and ball courts, as well as new installations of playground equipment and adult fitness facilities [37]. Renovations of a large number of parks took place in waves from 2017 to 2021, which presented a unique opportunity to implement a quasi-experiment to evaluate the association between park renovation and health outcomes within the communities living near these parks [38]. Utilizing data from the Physical Activity and Redesigned Community Spaces (PARCS) study, we investigated associations between the CPI park renovation intervention and changes in perceived stress among a sample of low-income predominantly Latino/a and Black adults in NYC. We also explored interactions between park use frequency and park renovation status to examine whether the association between park renovation and change in perceived stress was stronger among frequent park users, and if the association between frequent park use and change in perceived stress was stronger among those living near high-quality renovated parks compared to those living near lower-quality control parks.

## Methods

### Study design and setting

The PARCS study was a longitudinal, quasi-experimental prospective cohort study that took place in low-income neighborhoods in NYC between 2016 and 2022. The purpose of the study was to evaluate the health impacts of park redesign and renovation as a part of the CPI [37]. Full details on the study design are described elsewhere [38, 39]. The present analysis included participants drawn from 31 intervention parks and 21 socio-demographically matched control parks. Study sites, delineated as the area within a 0.3-mile radius (walking distance) of a public park, were eligible for inclusion if they had not received more than $250,000 in capital investments during the preceding 20 years, and if they met two of the following criteria:

- ≥ 20% of the population with income below the federal poverty line (high poverty).
- ≥ 25% population growth from 2000 to 2010 (high population growth).
- ≥ 110 residents per acre of land area (high population density).

### Intervention

The intervention was implemented by NYC Parks and involved the complete redesign and renovation of intervention parks, with a median capital investment of U.S. $3.9 million [IQR: $3.0 million – $5.0 million] per park site [40]. Both control and intervention park sites were eligible for CPI renovations; however, only the intervention sites were scheduled to be renovated during the study period. Renovations were tailored to specific community inputs and park conditions [41], and commonly included aesthetic enhancements, increased greenery and shade cover, new seating areas, upgraded play equipment, renovated sports facilities, additional comfort stations, and enhanced accessibility [39].

### Study population and recruitment

Recruitment and data collection methods for the PARCS study have been described in detail previously [38, 42]. Participants were eligible for recruitment into the study if they lived within a 0.3-mile radius of a study park, had no mobility issues, and spoke English, Spanish, or Chinese (the three most commonly spoken languages in NYC). Recruitment efforts primarily targeted residents living in public housing, but residents not living in public housing also qualified for the study if they lived in the neighborhood for at least two years and intended to stay in the neighborhood for at least four more years. Recruitment was done in partnership with the NYC Parks community outreach organization *Partnership for Parks*, where designated study ambassadors held regular engagement events in study neighborhoods, at study parks, and on social media [42]. Recruitment strategies were multifaceted and included both formal recruitment events as well as informal street-intercept recruitment [42]. When deemed eligible by study staff, residents filled out a survey on their mobile phone using a survey app or on paper if preferred by the participant. Participants were contacted by study ambassadors for follow-up by phone call, text message, e-mail, and/or an in-person visit to the participant’s residence. Various methods of contact, multiple follow-up attempts, and incentives ($50-$75 per participant for each survey completed) were employed to maximize retention in the study.

### Data collection

Outcomes and participant demographics were tracked over the course of the study using surveys administered pre- and post-renovation. Baseline data collection occurred at all study sites from 2016 to 2018, prior to the renovation of intervention parks. Intervention parks then closed for renovation and gradually reopened from 2017 to 2021. Follow-up data collection occurred at all intervention and matched control sites post-renovation, from 2018 to 2022. For the participants included in this study, median follow-up time was 25 months post-baseline for both the intervention and control groups. For the intervention group, the follow-up period corresponded to approximately seven months following the completion of renovations, on average.

### Measures

#### Outcome - perceived stress

Perceived stress was captured using the previously validated 14-item Perceived Stress Scale (PSS) [43]. The PSS is designed to measure the degree to which situations in an individual’s life are perceived as stressful. For each question, the survey asks about the frequency of stress-related thoughts and feelings, with answers on a five-point Likert scale ranging from “Never” (0) to “Very Often” (4). An overall PSS score was constructed as the sum of the 14 items (after reverse scoring some items), with higher PSS score representing higher perceived stress [range: 0–56].

#### Park use

Frequency of study park use was measured using a survey instrument previously validated by Veitch et al. [44]. This instrument assessed the frequency of park use in the past 30 days by asking participants how often they visited their study park: (1) daily, (2) 4-6 times per week, (3) 2-3 times per week, (4) once per week, (5) 2-3 times per month, (6) once per month, (7) less than once per month, or (8) no visit in the past 30 days. To maximize group sample sizes and to simplify analysis, participants were categorized into one of two groups based on their self-reported study park use frequency at follow-up: “high” (≥ once per week) vs. “low” (< once per week). By definition, the high park use group included participants who either reported an increase in park use post-renovation compared to pre-renovation (low to high) or those who reported high park use both pre- and post-renovation (high to high). The low park use group included participants who either reported a decrease in park use post-renovation compared to pre-renovation (high to low) or those who reported low park use both pre- and post-renovation (low to low). The cutoff of ≥ once per week as an indicator of frequent park use was selected based on previous studies that have demonstrated additional physical and mental health benefits for individuals who visit parks at least once per week [27, 29, 45, 46].

#### Sociodemographic variables

Sociodemographic variables included sex, race/ethnicity, age, annual household income, education, employment status, public housing, marital status, and children in household. Categories for sex included “Male” and “Female”. Age was categorized into three groups (“18-34y”, “35-49y”, and “50-78y”). Race and ethnicity were combined into a single composite measure, with values “Latino/a”, “Non-Latino/a Black”, and “Other or multiracial”. Education was categorized as “Some college or more” or “High school graduate or less”. Employment was categorized as “Employed or self-employed” or “Not employed”, which included individuals who considered themselves homemakers, students, retired, unemployed or unable to work. Public housing was an indicator of whether the participant lived in a NYC Housing Authority (NYCHA) residence with values of “NYCHA resident” and “Non-NYCHA resident”. Children in household referred to the number of people < 18y living in the participant’s household and was categorized as “No children”, “One child” or “Two or more children”. Marital status was categorized as “Never married”, “Married” or “Divorced, separated or widowed”.

### Statistical analysis

Participants who had completed the PSS survey items pre- and post-renovation as well as the park use frequency survey item post-renovation were included in this analysis. Multiple imputation by chained equations was used to impute a small number of missing baseline sociodemographic data (percent missing by variable: race/ethnicity– 1%, age– 1%, annual household income– 2%, education– 3%). Fifty imputed datasets were generated from the original data using the full set of baseline sociodemographic variables as predictor variables. The missing data points were then filled with the most common predicted values across the 50 imputed datasets. Convergence of the imputation algorithm was visually verified.

Descriptive statistics (mean (SD) and n (%)) and were produced for all sociodemographic variables at baseline in the sample overall and stratified by intervention status. Chi-squared tests and t-tests were used to screen for potential differences in the sociodemographic makeup of the intervention and control groups at baseline. Descriptive statistics (mean (SD)) were produced for the outcome (PSS score) pre- and post-renovation by intervention status.

A difference-in-difference (DID) approach for repeated measures using linear mixed-effects regression was used to measure the association between the CPI park renovation intervention and change over time in perceived stress at the individual participant level. DID is uniquely suited to estimate the effect of an intervention applied at the group level by comparing changes in the outcome over time in the exposed and unexposed groups while accounting for any permanent differences between them (e.g., unobserved park and neighborhood differences) and any common time trends affecting both groups [47].

The main linear regression model was fit to measure the association between park renovation and change in perceived stress in the overall sample. The main model included fixed effects for intervention status (intervention group vs. control group), time (pre-renovation vs. post-renovation), and the interaction between intervention status and time (the DID estimator). Stratified DID estimates were reported for any baseline sociodemographic variables exhibiting a significant (*p* < 0.05) three-way interaction with intervention status and time.

An additional linear regression model was fit using a three-way interaction term between intervention status, time, and study park use at follow-up to explore the associations between park renovation and change in perceived stress among participants with high vs. low park use frequency. The same model was also used to explore associations between park use frequency and change in perceived stress among residents living near renovated parks vs. control parks. Estimates for this three-way interaction model were presented for the overall sample, as well as stratified by age group (18-34y, 35-49y, 50-78y).

All models were adjusted for baseline sociodemographic variables found to be imbalanced between the intervention and control groups. Within-participant and within-park correlations were accounted for by incorporating random effects into the models. For all models, we present the model-estimated within-group change in mean PSS scores post-renovation minus pre-renovation and between-group difference estimates with their respective 95% confidence intervals. For DID estimators, we also report exact p-values. A two-tailed α = 0.05 was used for all significance tests. Assumptions for linear mixed-effects regression were verified using residual plots.

Data cleaning and analyses were performed in R software version 4.4.1 (https://www.R-project.org/). Multiple imputation was performed using the *mice* package [48]. Linear mixed-effects regression models were fit using the *lme4* and *lmerTest* packages [49, 50]. Contrasts were extracted from fitted interaction models using the *emmeans* package [51]. Data visualizations were created using *ggplot2* [52].

### Sensitivity analysis

Most participants (89%, *n* = 279) completed post-renovation PSS surveys before the COVID-19 pandemic began in March 2020, while 11% (*n* = 34) did so afterward. Surveys conducted elsewhere during the pandemic show evidence of a sharp rise in perceived stress experienced collectively across the U.S [1]. and in NYC [14] after the start of the pandemic. The COVID-19 pandemic might have differentially impacted the responses given by participants who completed their post-renovation PSS surveys during and after March 2020; thus, we performed a sensitivity analysis excluding these 34 participants to compare with the full sample results.

An additional sensitivity analysis was performed after varying the cut points for high vs. low study park use from “≥ once per week” vs. “< once per week” to: (variation 1) “> once per week” vs. “≤ once per week” and (variation 2) “> once per month” vs. “≤ once per month”.

### Ethics

This study was approved by the City University of New York Institutional Review Board (#2016 − 0248). All participants provided written informed consent prior to participation in the study.

## Results

### Sample characteristics

A total of 313 adult participants enrolled in the PARCS study had completed the PSS survey at both baseline and follow-up, and had completed the park use questionnaire at follow-up. This included 162 participants living near 31 parks that received the CPI park renovation intervention and 151 participants living near 21 control parks. The median number of participants per park site was 6 [IQR: 4–8]. 

PSS scores were similar between intervention and control groups at baseline, with an overall mean (SD) of 23.83 (7.34), corresponding to “moderate” stress [53]. Study participants were majority female (86%), Latino/a (47%) or non-Latino/a Black (34%), with a mean age of 41 years (SD = 12 years) (Table  1 ). Forty-six percent of study participants came from households that made less than $20,000 annually, 45% had a high school diploma or less education, and 50% were employed or self-employed. Fifty percent of study participants resided in public housing (NYCHA residences). A large proportion of participants were never married (47%), with 30% married, and 23% divorced, widowed or separated. Most participants had at least one child (< 18y) living in the household, with 29% reporting one child and 43% reporting two or more children.

**Table 1:** Characteristics of adult PARCS study participants with complete PSS and park use frequency questionnaires

**Characteristic**	**Overall ** *n* = 313^1^	**Intervention ** *n* = 162^1^	**Control ** *n* = 151^1^	***p*** **-value** ^**2**^
PSS Score at Baseline	23.83 (7.34)	23.25 (6.85)	24.45 (7.81)	0.20
Sex				0.80
Female	268 (86%)	138 (85%)	130 (86%)	
Male	45 (14%)	24 (15%)	21 (14%)	
Race/Ethnicity				0.40
Latino/a	147 (47%)	78 (48%)	69 (46%)	
Non-Latino/a Black	105 (34%)	57 (35%)	48 (32%)	
Other or multiracial	61 (19%)	27 (17%)	34 (23%)	
Age	41 (12)	41 (13)	41 (11)	0.60
Age Group				0.80
18-34y	104 (33%)	54 (33%)	50 (33%)	
35-49y	128 (41%)	64 (40%)	64 (42%)	
50-78y	81 (26%)	44 (27%)	37 (25%)	
Annual Household Income				0.60
Less than $20,000	144 (46%)	72 (44%)	72 (48%)	
$20,000 or more	169 (54%)	90 (56%)	79 (52%)	
Education				0.053
High school graduate or less	142 (45%)	82 (51%)	60 (40%)	
Some college or more	171 (55%)	80 (49%)	91 (60%)	
Employment Status				0.50
Employed or self-employed	158 (50%)	85 (52%)	73 (48%)	
Not employed	155 (50%)	77 (48%)	78 (52%)	
Public Housing				0.080
Non-NYCHA resident	157 (50%)	89 (55%)	68 (45%)	
NYCHA resident	156 (50%)	73 (45%)	83 (55%)	
Marital Status				0.080
Never married	148 (47%)	80 (49%)	68 (45%)	
Married	94 (30%)	40 (25%)	54 (36%)	
Divorced, separated or widowed	71 (23%)	42 (26%)	29 (19%)	
Children in Household				0.30
No children	88 (28%)	49 (30%)	39 (26%)	
One child	91 (29%)	50 (31%)	41 (27%)	
Two or more children	134 (43%)	63 (39%)	71 (47%)	
Study Park Use at Follow-Up				0.40
High (≥ Once per week)	173 (55%)	93 (57%)	80 (53%)	
Low (< Once per week)	140 (45%)	69 (43%)	71 (47%)	

*Abbreviations*: *PARCS *Physical Activity and Redesigned Community Spaces, *PSS *Perceived Stress Scale, *NYCHA *New York City Housing Authority

^1^Mean (SD); *n* (%)

^2^Pearson's Chi-squared test; Welch Two Sample t-test

Sociodemographic covariates were well-balanced overall between the control and intervention groups, with marginally significant differences between the intervention and control groups on education (*p* = 0.053), public housing (*p* = 0.080), and marital status (*p* = 0.080) at baseline (Table 1). Compared to the control group, the intervention group showed slightly lower educational attainment (51% high school graduate or less in intervention group vs. 40% in control group), slightly lower residence in public housing (45% NYCHA residents in intervention group vs. 55% in control group), and slightly fewer married (25% in intervention group vs. 36% in control group) and more divorced, separated or widowed participants (26% in intervention group vs. 19% in control group). Study park use at follow-up (post-renovation) was similar between the two groups, with 57% of participants in the intervention group having visited the study park ≥ once per week compared to 53% of participants in the control group.

### Changes in perceived stress over time by intervention status

**Fig. 1:**
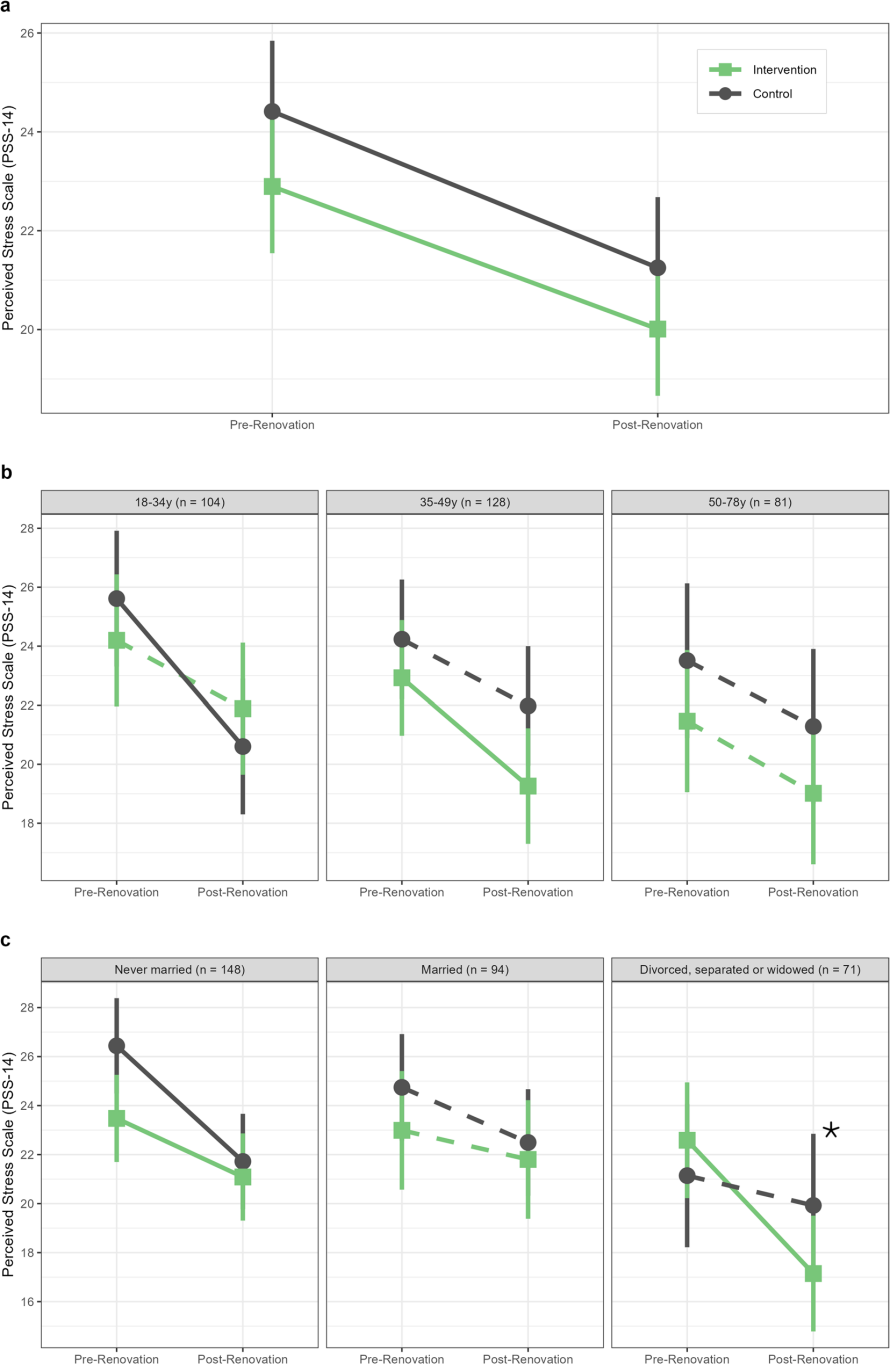
Mean PSS scores among adult PARCS study participants before and after CPI park renovation intervention in (**a**) overall sample (**b**) stratified by age at baseline and (**c**) stratified by marital status at baseline. Means by intervention group (green square = intervention, grey circle = control) and 95% confidence intervals (vertical bars) were estimated using a linear mixed effects regression models adjusted for education, public housing, and marital status at baseline. Solid lines indicate significant (*p *< 0.05) change over time in mean PSS score within a given group, while dashed lines indicate non-significant change over time. The asterisk indicates a significant DID estimate among divorced, separated or widowed participants. Abbreviations– PSS: Perceived Stress Scale; PARCS: Physical Activity and Redesigned Community Spaces; CPI: Community Parks Initiative; DID: Difference-in-Difference

In the overall sample, there was no difference in change in perceived stress between participants living near parks receiving renovations compared to those living near control parks [DID = 0.28 (95% CI −1.48, 2.03), *p* = 0.75] (Table 2; Fig. 1a). Although the changes over time in perceived stress were similar between the two groups, both intervention participants and control participants experienced a significant decrease in average PSS scores in the post-renovation period compared to the pre-renovation period [intervention group change = −2.88 (95% CI −4.48, −1.29), control group change = −3.16 (95% CI −4.82, −1.51)].

**Table 2:** Changes in perceived stress (as measured by PSS score) over time among adult PARCS study participants in intervention vs. control groups in overall sample and stratified by age and marital status at baseline

	**Intervention (** ***n*** ** = 162)**			**Control (** ***n*** ** = 151)**			**Difference-in-Differences**	
	**Pre- Renovation** ^1^	**Post-Renovation** ^1^	**Change ** **(95% CI)** ^2^	**Pre-Renovation** ^1^	**Post-Renovation** ^1^	**Change ** **(95% CI)** ^2^	**DID Estimator (95% CI)** ^**2,3**^	***p*** **-value**
Overall Sample	23.25 (6.85)	20.37 (8.23)	−2.88 (−4.48, −1.29)	24.45 (7.81)	21.28 (8.08)	−3.16 (−4.82, −1.51)	0.28 (−1.48, 2.03)	0.75
Age at Baseline								
18-34y	24.39 (6.84)	22.08 (8.91)	−2.32 (−5.08, 0.45)	25.93 (6.84)	20.92 (9.12)	−5.01 (−7.89, −2.14)	2.70 (−0.34, 5.73)	0.082
35-49y	23.06 (6.34)	19.39 (7.66)	−3.67 (−6.21, −1.13)	24.13 (8.12)	21.87 (7.46)	−2.26 (−4.80, 0.28)	−1.41 (−4.14, 1.33)	0.31
50-78y	22.15 (7.49)	19.70 (8.04)	−2.45 (−5.51, 0.62)	22.99 (8.34)	20.76 (7.77)	−2.23 (−5.57, 1.11)	−0.22 (−3.67, 3.23)	0.90
Marital Status at Baseline								
Never married	23.76 (7.16)	21.37 (8.21)	−2.39 (−4.64, −0.14)	26.36 (8.58)	21.64 (8.61)	−4.72 (−7.16, −2.28)	2.33 (−0.20, 4.85)	0.071
Married	22.75 (5.99)	21.57 (6.67)	−1.19 (−4.37, 1.99)	24.08 (6.11)	21.83 (7.27)	−2.25 (−4.98, 0.49)	1.06 (−2.13, 4.26)	0.51
Divorced, separated or widowed	22.77 (7.09)	17.33 (9.01)	−5.44 (−8.54, −2.34)	20.64 (7.46)	19.43 (8.24)	−1.22 (−4.95, 2.52)	−4.22 (−7.92, −0.53)	0.025

*Abbreviations*: *PSS *Perceived Stress Scale, *PARCS *Physical Activity and Redesigned Community Spaces, *DID *Difference-in-Difference

^1^Mean (SD)

^2^Estimated using linear mixed effects regression models adjusted for education, public housing, and marital status at baseline

^3^The DID estimator represents the difference in change in mean PSS score in the intervention group minus the control group

### Changes in perceived stress over time by intervention status and baseline demographics

We found significant three-way interactions between intervention status, time, and age at baseline (*p* = 0.049) and intervention status, time, and marital status at baseline (*p* = 0.004), indicating potential differences in perceived stress change over time by age and marital status at baseline. No significant interactions were found for the remaining baseline demographic variables (sex, race/ethnicity, annual household income, education, employment status, public housing, and children in household).

####  Age

No significant DID effects for park renovation were found in any of the three age groups [18-34y DID = 2.70 (95% CI −0.34, 5.73), *p* = 0.082; 35-49y DID = −1.41 (95% CI −4.14, 1.33), *p* = 0.31; 50-78y DID = −0.22 (95% CI −3.67, 3.23), *p* = 0.90] (Table 2; Fig. 1b). However, among the 18-34y group, only the control group had a significant decrease in perceived stress over time [change = −5.01 (95% CI −7.89, −2.14)], while the intervention group showed no significant change in perceived stress over time [change = −2.32 (95% CI −5.08, 0.45)]. Among the 35-49y group, only the intervention group had a significant decrease in perceived stress over time [change = −3.67 (95% CI −6.21, −1.13)], while the control group showed no significant change in perceived stress over time [change = −2.26 (95% CI −4.80, 0.28)]. Among 50-78y olds, neither group experienced a significant change in perceived stress over time [intervention change = −2.45 (95% CI −5.51, 0.62), control change = −2.23 (95% CI −5.57, 1.11)].

#### Marital status

Among participants divorced, separated or widowed at baseline, those living near parks receiving renovations experienced a significantly larger decrease in perceived stress over the study period compared to those living near control parks [DID = −4.22 (95% CI −7.92, −0.53), *p* = 0.025] (Table 2; Fig. 1c). For the other two groups, changes over time in perceived stress were similar between intervention and control groups [never married DID = 2.33 (95% CI −0.20, 4.85), *p* = 0.071; married DID = 1.06 (95% CI −2.13, 4.26), *p* = 0.51].

### Changes in perceived stress over time by intervention status and study park use at follow-up

We also found significant three-way interactions between intervention status, time, and park use frequency at follow-up in the overall sample (*p* = 0.027) and among participants aged 35-49y (*p* = 0.025) (Table 3).

**Table 3:** Changes in perceived stress (as measured by PSS score) over time among adult PARCS study participants by intervention status and study park use at follow-up, in overall sample and stratified by age at baseline

**Overall Sample**	**Intervention**	**Control**	**Difference **
	**(*n* = 162)**^**1**^	**(*n* = 151)**^**1**^	**(Intervention - Control)**^2^
Study Park Use at Follow-Up			
High (≥ Once per week) (*n* = 173)	−4.13 (−6.22, −2.03)	−2.67 (−4.93, −0.41)	−1.46 (−3.81, 0.89)
Low (< Once per week) (*n* = 140)	−1.21 (−3.64, 1.22)	−3.72 (−6.12, −1.32)	2.51 (−0.09, 5.11)
Difference (High - Low)^3^	−2.92 (−5.36, −0.47)	1.05 (−1.46, 3.56)	3-way interaction^4^ *p* = 0.027
**Age 18-34y**	**Intervention**	**Control**	**Difference **
	**(*n*= 54)**^**1**^	**(*n*=50)**^**1**^	**(Intervention-Control)**^**2**^
Study Park Use at Follow-Up			
High (≥ Once per week) (*n* = 52)	−4.71 (−7.79, −1.64)	−4.76 (−7.72, −1.80)	0.04 (−4.23, 4.31)
Low (< Once per week) (*n* = 52)	−0.25 (−3.10, 2.61)	−5.31 (−8.52, −2.10)	5.06 (0.77, 9.36)
Difference (High - Low)^3^	−4.47 (−8.66, −0.27)	0.56 (−3.81, 4.92)	3-way interaction^4^ *p* = 0.10
**Age35**-**49y**	**Intervention**	**Control**	**Difference**
	**(*n*=64)**^**1**^	**(*n*=64)**^**1**^	**(Intervention-Control)**^**2**^
Study Park Use at Follow-Up			
High (≥ Once per week) (*n* = 65)	−5.26 (−7.93, −2.58)	−0.80 (−3.51, 1.92)	−4.46 (−8.28, −0.64)
Low (< Once per week) (*n* = 63)	−1.98 (−4.74, 0.79)	−3.73 (−6.45, −1.01)	1.75 (−2.13, 5.63)
Difference (High - Low)^3^	−3.28 (−7.13, 0.57)	2.93 (−0.92, 6.78)	3-way interaction^4^ *p* = 0.025
**Age50-78y**	**Intervention**	**Control**	**Difference**
	**(*n*=44)**^**1**^	**(*n*=37)**^**1**^	**(Intervention-Control)**^**2**^
Study Park Use at Follow-Up			
High (≥ Once per week) (n = 56)	−2.64 (−5.24, −0.04)	−2.84 (−6.20, 0.52)	0.20 (−4.05, 4.44)
Low (< Once per week) (n = 25)	−1.68 (−6.80, 3.45)	−1.42 (−5.27, 2.42)	−0.25 (−6.66, 6.15)
Difference (High - Low)^3^	−0.97 (−6.72, 4.78)	−1.42 (−6.52, 3.68)	3-way interaction^4^ *p* = 0.91

Estimates in table are from linear mixed effects regression models adjusted for education, public housing, and marital status at baseline

^1^Mean change in PSS (post-renovation mean– pre-renovation mean) and 95% CI

^2^Difference in mean change in PSS between intervention and control groups and 95% CI

^3^Difference in mean change in PSS between high and low park use groups and 95% CI

^4^*p*-value is for 3-way interaction between intervention status * time * study park use at follow-up

**Fig. 2:**
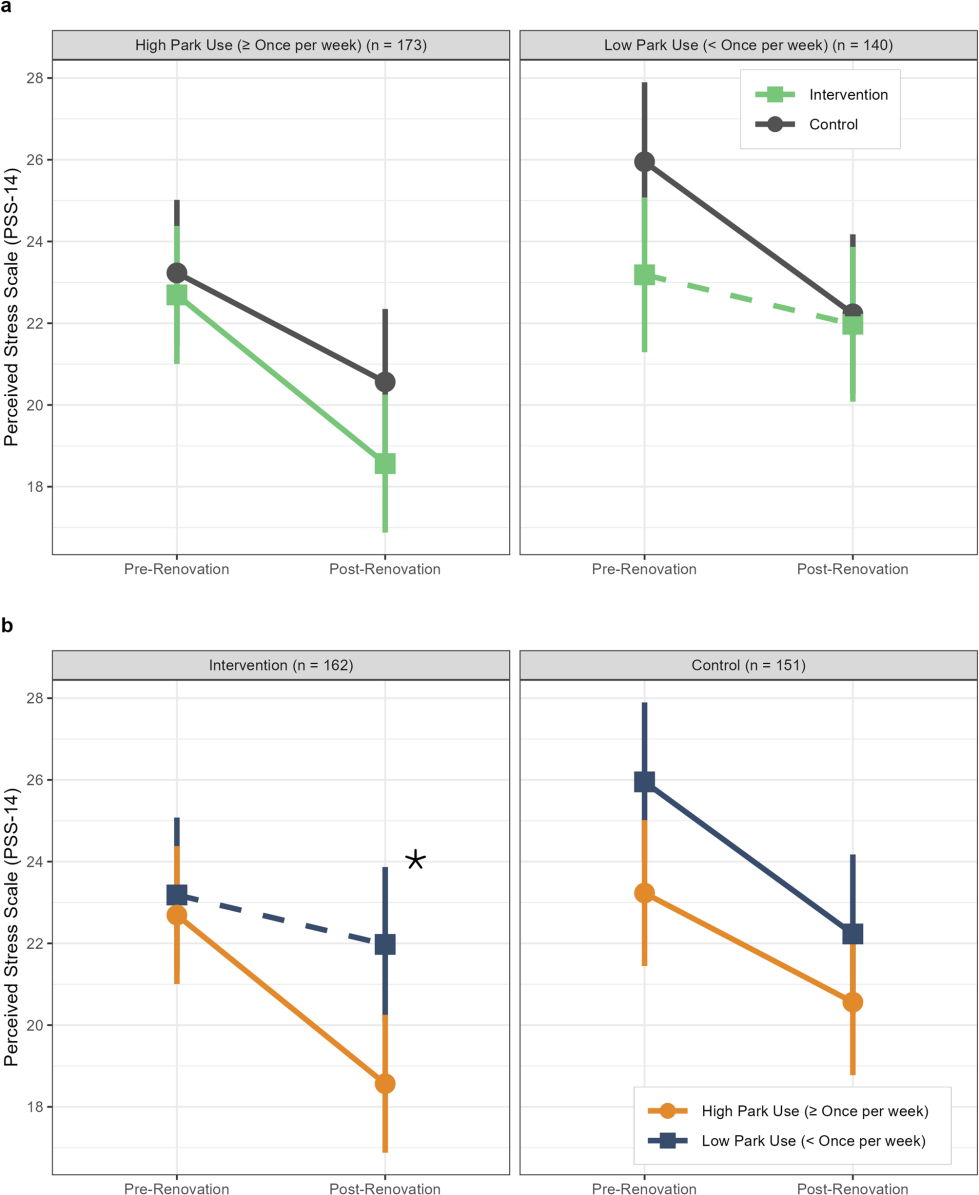
Mean PSS scores among adult PARCS study participants before and after CPI park renovation (**a**) in intervention vs. control groups stratified by frequency of study park use at follow up, and (**b**) for participants with high park use (≥ once per week) vs. low park use (< once per week) stratified by intervention status. Means by park use frequency at follow-up and intervention group and 95% confidence intervals (vertical bars) were estimated using a linear mixed effects regression model adjusted for education, public housing, and marital status at baseline. In (**a**), the results are stratified by park use frequency at follow-up, with intervention group means represented by green squares, and control group means represented by grey circles. In (**b**), the same results are stratified by intervention status, with mean PSS for high park use represented by blue squares, and mean PSS for low park use represented by orange circles. Solid lines indicate significant (*p *< 0.05) change over time in mean PSS score within a given group, while dashed lines indicate non-significant change over time. The asterisk symbol indicates a significantly larger decrease in PSS over time among participants with high park use compared to those with low park use within the intervention group. Abbreviations– PSS: Perceived Stress Scale; PARCS: Physical Activity and Redesigned Community Spaces; CPI: Community Parks Initiative

**Fig. 3:**
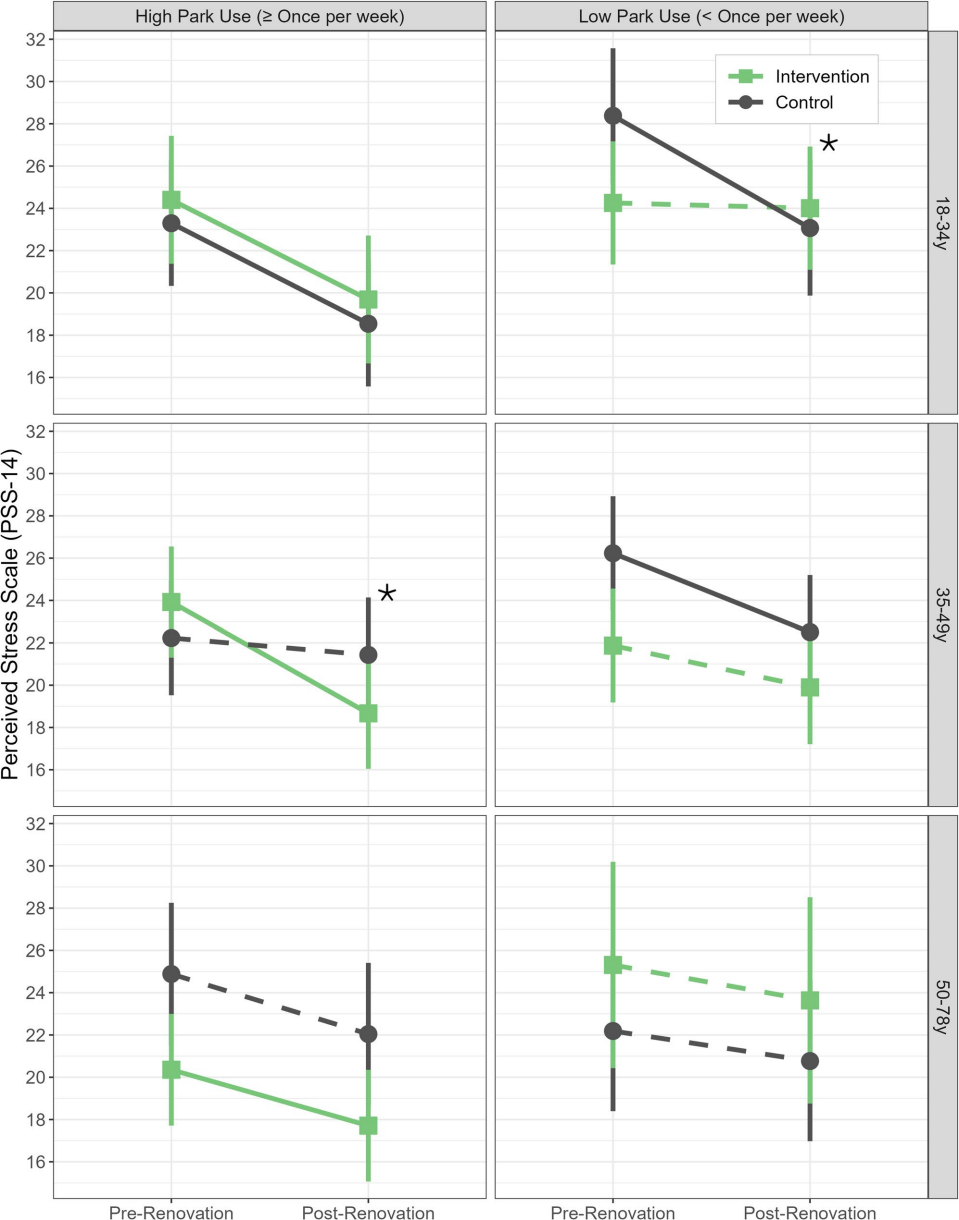
Mean PSS scores among adult PARCS study participants before and after CPI park renovation intervention stratified by age at baseline and study park use at follow-up. Means by intervention group (green square = intervention, grey circle = control) and 95% confidence intervals (vertical bars) were estimated using a linear mixed effects regression models adjusted for education, public housing, and marital status at baseline. Solid lines indicate significant (*p *< 0.05) change over time in mean PSS score within a given group, while dashed lines indicate non-significant change over time. The asterisks indicate significant differences between intervention and control groups in change in PSS within a given subgroup. Abbreviations– PSS: Perceived Stress Scale; PARCS: Physical Activity and Redesigned Community Spaces; CPI: Community Parks Initiative

In the overall sample, the interaction was driven by a significantly larger decrease in perceived stress among participants with high park use in the intervention group [change (post– pre) = −4.13 (95% CI −6.22, −2.03)] compared to participants with low park use in the intervention group [change (post– pre) = −1.21 (95% CI −3.64, 1.22)] [difference in change (high park use– low park use) = −2.92 (95% CI −5.36, −0.47)] (Table 3; Fig. 2). There was no association between park use frequency and change in perceived stress in either the control group [difference in change (high park use– low park use) = 1.05 (95% CI −1.46, 3.56)] (Table 3; Fig. 2) or the overall sample [difference in change (high park use– low park use) = −0.97 (−2.73, 0.79)] (Supplementary Material 1– Table 1). Among participants aged 35-49y, the interaction was driven by a significantly larger decrease in perceived stress among those with high park use in the intervention group [change (post– pre) = −5.26 (95% CI −7.93, −2.58)] compared to those with high park use in the control group [change (post– pre) = −0.80 (95% CI −3.51, 1.92)] [difference in change (intervention - control) = −4.46 (95% CI −8.28, −0.64)] (Table 3; Fig. 3). In the other age groups (18-34y and 50-78y), there was no difference in change in perceived stress between intervention and control groups among individuals with high or low study park use.

### Model validation and sensitivity analyses

All fitted mixed-effects regression models satisfied the assumptions for linear regression (residuals were normally distributed and homoscedastic– see residual plots for main model in Supplementary Material 1– Fig. 1). A single influential outlier was observed (Cook’s D > 0.5) which after removal did not change the overall conclusions drawn from the model estimates (Supplementary Material 1– Table 2). Excluding *n* = 34 participants with post-renovation PSS surveys completed during or after March 2020 also did not change the overall conclusions drawn from the model estimates (Supplementary Material 1– Table 3). Model estimates also remained unchanged after additionally adjusting for study park use at follow-up (Supplementary Material 1– Table 4).

After varying the cut points for study park use to “> once per week” vs. “≤ once per week” (Supplementary Material 1– Table 5) and “> once per month” vs. “≤ once per month” (Supplementary Material 1– Table 6), the association between study park use and change in perceived stress remained in the same direction, with only the intervention group showing a larger decrease in perceived stress among those with high study park use compared to those with low study park use. However, the strength of this association was slightly attenuated, with the three-way interaction terms between intervention status, time, and study park use becoming non-significant in both scenarios.

## Discussion

This study represents one of the first and largest park quality improvement studies to-date, evaluating the health impacts of park redesign and renovation in 31 intervention parks with 21 socio-demographically-matched control parks in NYC– the second largest urban center in North America and one of the most global cities in the region with 38% of its population born outside the U.S [54]. In this evaluation, we found no direct overall association between park renovation and change in perceived stress at the individual level in a sample of low-income, predominantly Latino/a and Black adults. However, we did find that frequent (≥ once per week) park use was significantly negatively associated with change in perceived stress in the intervention group but not in the control group. Our findings suggest that while park renovation alone may not have a direct impact on stress reduction in all groups, frequent use of *high-quality* renovated parks may be associated with greater mental health benefits.

To date, few intervention studies have measured the impact of park renovations on individual-level health outcomes. This is the first study, to our knowledge, to evaluate individual-level associations between park renovation and mental health, as most park renovation intervention studies in the past have focused on park use and physical activity-related outcomes [55, 56], with a small number of studies examining well-being outcomes measured by direct observation or repeated cross-sectional surveys [57–59]. Furthermore, park improvement efforts described in previous studies have generally been geographically limited, focusing on a small number (< 10) of urban or suburban parks. Compared to previous studies, this study is further strengthened by using the DID analytic approach which controls for the effects of unobserved confounders and time trends affecting both the intervention and control groups.

Among participants living near parks receiving renovations as part of the CPI, we found that those who frequently (≥ once per week) used the renovated parks had a significantly larger drop in perceived stress over the study period compared to those who used the renovated parks < once per week. This finding is in line with previous observational studies that have demonstrated a dose-response effect of frequent (weekly) use of urban greenspaces and parks on mental health and wellbeing [27, 29]. Interestingly, this same relationship was not apparent among participants living near control parks, which suggests that park quality may play an important moderating role in the relationship between park use frequency and the mental health benefits of parks. In other words, high quality park spaces may be an important pre-condition for the health benefits of frequent park use to take place.

Population-based longitudinal and cross-sectional studies have repeatedly demonstrated that perceived stress decreases with age, and that young adults (< 35y) experience the highest stress exposure and negative responses to stress [60–62]. In our study, both the intervention and control groups overall experienced a significant decrease in perceived stress over the study period, which is likely attributable to this aging effect. Across age groups at baseline, we observed an overall decreasing trend in perceived stress with increasing age, although we observed different trends within age groups in the magnitude of change in perceived stress over time by intervention status. Participants over 50 (50-78y) did not experience a significant post-renovation decrease in perceived stress in either the control or intervention group. In the middle-aged (35-49y) group, only the intervention participants experienced a significant decrease in perceived stress post-renovation, while in the younger (18-34y) group only the control participants experienced a significant decrease in perceived stress. Furthermore, only frequent park users in the middle-aged (35-49y) group experienced a significant treatment effect of park renovation on reduced stress. Differences in park use behaviors by age may offer some explanation for these differential findings. Middle-aged individuals, for instance, might utilize renovated parks in ways that are more conducive to stress reduction, such as engaging in relaxation and physical activities. More research is needed to fully understand how age-related differences in park use behaviors contribute to stress reduction, and how targeted park renovations might support various stress-reduction pathways.

We also found a significant treatment effect of park renovation on stress reduction within the divorced, separated or widowed group. Within this group, intervention participants experienced a more than four-point larger drop in PSS scores post-renovation compared to control participants. This finding contributes to a growing body of literature demonstrating parks and greenspaces as potential mental health buffers during and after traumatic experiences such as the loss of a loved one, violence or abuse, or the COVID-19 pandemic [21, 63]. One recent study linked exposure to greenspace to post-traumatic stress disorder (PTSD) recovery trajectories, where trauma survivors with more access to greenspace had better resiliency to PTSD after trauma [63]. Renovated parks in particular may provide respite for individuals going through or recovering from traumatic experiences by providing safe, high-quality, and aesthetic spaces to connect to nature, self, family, friends and community [64].

This study has several limitations. First, the PSS-14 relies on self-reported, subjective measures of perceived stress rather than objective measures of stressor exposure and may therefore be open to recall bias and other biases common to self-reported survey instruments. Second, the participants included in the study represent a convenience sample and may not represent the underlying populations in the study catchment areas, therefore generalizability of the study findings may be limited. Third, lack of study power may have limited our ability to identify statistically significant findings more broadly. Fourth, we did not track changes in programming and events offered at parks, so it is possible that the lack of intervention effect could be due, in part, to lack of park programming. More research is needed to understand the role of park programming in the context of high-quality renovated park spaces in modifying park use behaviors and mental health outcomes. Finally, only one round of pre-renovation outcomes data was collected, which limited our ability to empirically evaluate the parallel trends assumption for DID analysis. However, we examined trends over time in the sociodemographic composition (e.g., % Black, % Latino/a, % in poverty, % with a Bachelor’s degree or higher, % disabled, % over 50 years of age) of the neighborhoods surrounding intervention and control parks at three time points prior to the CPI park renovation intervention (2013, 2015, and 2017) using five-year estimates at the census block level from the American Community Survey [65], and we found no differences in the rate of change in sociodemographic composition of intervention and control sites prior to the study period.

## Conclusions

In conclusion, no direct overall association was found between citywide park renovation and change in perceived stress in low-income, predominantly Latino/a and Black neighborhoods in NYC. However, a direct association between park renovation and decreased perceived stress was found in certain subgroups (divorced/separated/widowed participants, and middle-aged participants with frequent park use). In addition, we found a significant negative association between park use frequency and change in perceived stress within CPI-renovated parks, which was not observed in control parks, suggesting that high-quality parks may be an important pre-condition for the mental health benefits of frequent park use. High-quality renovated park spaces may play an important role in facilitating the stress-reducing benefits of frequent park use.

## Supplementary Information

Supplementary Material 1.

## Data Availability

The datasets used and/or analyzed during the current study are available from the corresponding author on reasonable request.

## Abbreviations

CPI: Community Parks Initiative
DID: Difference–in–difference
NYC: New York City
NYCHA: New York City Housing Authority
PARCS: Physical Activity and Redesigned Community Spaces
PSS: Perceived Stress Scale
