# Supplementary material for: Citywide park renovations and changes in perceived stress: a quasi-experimental study among low-income communities in New York City
Source: BMC Public Health. 2025 Jul 19;25:2515. doi: 10.1186/s12889-025-23639-7 (PMC12275236; doi:10.1186/s12889-025-23639-7)
Supplement: Supplementary file 1 — Supplementary Material 1. [file 12889_2025_23639_MOESM1_ESM.docx]

**Citywide park renovations and changes in perceived stress: a quasi-experimental study among low-income communities in New York City**

Supplementary Material 1

Authors: Rachel L. Thompson, Katarzyna E. Wyka, Kelly R. Evenson, Lorna E. Thorpe, Glen D. Johnson, Brian T. Pavilonis, and Terry T.-K. Huang

**Table of Contents**

**Figure 1. Residual plots validating assumptions for linear regression**

**Table 1. Overall association between study park use and change in perceived stress among adult PARCS study participants**

**Table 2. Changes in perceived stress over time among adult PARCS study participants in intervention vs. control groups after excluding single influential outlier**

**Table 3. Changes in perceived stress over time among adult PARCS study participants in intervention vs. control groups after excluding participants with follow-up surveys collected during and after March 2020 (COVID-19 pandemic)**

**Table 4. Changes in perceived stress over time among adult PARCS study participants in intervention vs. control groups after additionally adjusting for study park use**

**Table 5. Changes in perceived stress over time among adult PARCS study participants by intervention status and study park use at follow-up, with high study park use defined as “> Once per week” and low study park use defined as “≤ Once per week”**

**Table 6. Changes in perceived stress over time among adult PARCS study participants by intervention status and study park use at follow-up, with high study park use defined as “> Once per month” and low study park use defined as “≤ Once per month”**

**Figure 1. Residual plots validating assumptions for linear regression (from main model)**


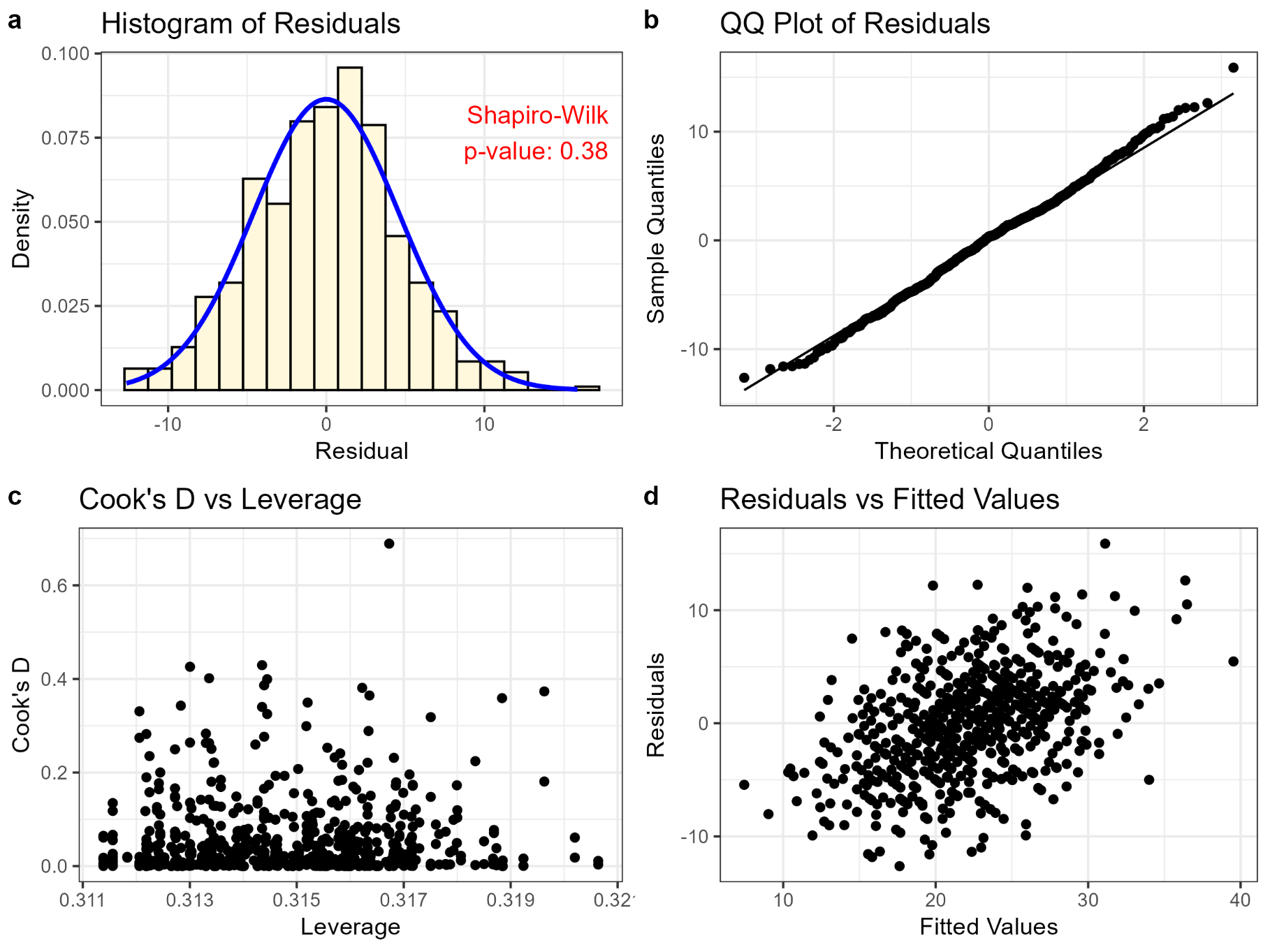


**Table 1. Overall association between study park use and change in perceived stress among adult PARCS study participants**

| **High Study Park Use at Follow-Up**  **(≥ Once per week)**  **(n = 173)** | | | **Low Study Park Use at Follow-Up**  **(< Once per week)**  **(n = 140)** | | | **Difference**  **(High – Low)** | |
| --- | --- | --- | --- | --- | --- | --- | --- |
| **Pre-Renovation**^1^ | **Post-Renovation**^1^ | **Change**  **(95% CI)** ^2^ | **Pre-Renovation**^1^ | **Post-Renovation**^1^ | **Change**  **(95% CI)** ^2^ | **Difference Estimate**  **(95% CI)^2,3^** | **p-value** |
| 23.07 (6.97) | 19.61 (7.54) | -3.45 (-5.00, -1.91) | 24.77 (7.69) | 22.29 (8.67) | -2.48 (-4.20, -0.77) | -0.97 (-2.73, 0.79) | 0.28 |

^1^Mean (SD)

^2^Estimated using linear mixed effects regression models

^3^Difference in change in mean PSS score among individuals with high study park use at follow-up minus the change in mean PSS score among individuals with low study park use at follow-up

Abbreviations – PSS: Perceived Stress Scale; PARCS: Physical Activity and Redesigned Community Spaces; DID: Difference-in-Difference

**Table 2. Changes in perceived stress over time among adult PARCS study participants in intervention vs. control groups after excluding single influential outlier***

|  | **Intervention**  **(n = 161)** | | | **Control**  **(n = 151)** | | | **Difference-in-Differences** | |
| --- | --- | --- | --- | --- | --- | --- | --- | --- |
|  | **Pre-Renovation**^1^ | **Post-Renovation**^1^ | **Change**  **(95% CI)** ^2^ | **Pre-Renovation**^1^ | **Post-Renovation**^1^ | **Change**  **(95% CI)** ^2^ | **DID Estimator (95% CI)^2,3^** | **p-value** |
| **Overall Sample** | 23.22 (6.85) | 20.20 (7.99) | -3.01 (-4.60, -1.43) | 24.45 (7.81) | 21.28 (8.08) | -3.16 (-4.80, -1.52) | 0.15 (-1.59, 1.89) | 0.87 |
| **Age at Baseline** |  |  |  |  |  |  |  |  |
| 18-34y | 24.39 (6.84) | 22.08 (8.91) | -2.32 (-5.05, 0.42) | 25.93 (6.84) | 20.92 (9.12) | -5.01 (-7.85, -2.17) | 2.70 (-0.31, 5.70) | 0.078 |
| 35-49y | 22.96 (6.34) | 18.95 (6.86) | -4.01 (-6.54, -1.48) | 24.13 (8.12) | 21.87 (7.46) | -2.26 (-4.77, 0.25) | -1.75 (-4.47, 0.97) | 0.21 |
| 50-78y | 22.15 (7.49) | 19.70 (8.04) | -2.45 (-5.48, 0.58) | 22.99 (8.34) | 20.76 (7.77) | -2.23 (-5.53, 1.08) | -0.22 (-3.63, 3.20) | 0.90 |
| **Marital Status at Baseline** |  |  |  |  |  |  |  |  |
| Never married | 23.69 (7.18) | 21.04 (7.73) | -2.65 (-4.89, -0.41) | 26.36 (8.58) | 21.64 (8.61) | -4.72 (-7.13, -2.31) | 2.07 (-0.44, 4.58) | 0.11 |
| Married | 22.75 (5.99) | 21.57 (6.67) | -1.19 (-4.34, 1.96) | 24.08 (6.11) | 21.83 (7.27) | -2.25 (-4.96, 0.46) | 1.06 (-2.10, 4.22) | 0.51 |
| Divorced, separated, or  widowed | 22.77 (7.09) | 17.33 (9.01) | -5.44 (-8.51, -2.37) | 20.64 (7.46) | 19.43 (8.24) | -1.22 (-4.91, 2.48) | -4.22 (-7.88, -0.56) | 0.024 |
| **Study Park Use at Follow-Up** |  |  |  |  |  |  |  |  |
| High (≥ Once per week) | 23.14 (6.93) | 19.01 (7.41) | -4.13 (-6.21, -2.05) | 22.98 (7.07) | 20.31 (7.67) | -2.67 (-4.91, -0.43) | -1.46 (-3.79, 0.87) | 0.22 |
| Low (< Once per week) | 23.32 (6.79) | 21.83 (8.50) | -1.49 (-3.92, 0.94) | 26.10 (8.31) | 22.38 (8.44) | -3.72 (-6.10, -1.34) | 2.23 (-0.36, 4.82) | 0.092 |
| ^1^Mean (SD)  ^2^Estimated using linear mixed effects regression models adjusted for education, public housing, and marital status at baseline  ^3^The DID estimator represents the difference in change in mean PSS score in the intervention group minus the control group  *The outlier was identified based on Cook’s D > 0.5  Abbreviations – PSS: Perceived Stress Scale; PARCS: Physical Activity and Redesigned Community Spaces; DID: Difference-in-Difference | | | | | | | | |

**Table 3. Changes in perceived stress over time among adult PARCS study participants in intervention vs. control groups after excluding participants with follow-up surveys collected during and after March 2020 (COVID-19 pandemic)**

|  | **Intervention**  **(n = 134)** | | | **Control**  **(n = 145)** | | | **Difference-in-Differences** | |
| --- | --- | --- | --- | --- | --- | --- | --- | --- |
|  | **Pre-Renovation**^1^ | **Post-Renovation**^1^ | **Change**  **(95% CI)** ^2^ | **Pre-Renovation**^1^ | **Post-Renovation**^1^ | **Change**  **(95% CI)** ^2^ | **DID Estimator (95% CI)^2,3^** | **p-value** |
| **Overall Sample** | 23.28 (6.59) | 19.95 (8.22) | -3.33 (-5.07, -1.59) | 24.41 (7.84) | 21.18 (8.16) | -3.23 (-4.90, -1.56) | -0.10 (-1.93, 1.74) | 0.92 |
| **Age at Baseline** |  |  |  |  |  |  |  |  |
| 18-34y | 24.38 (6.24) | 21.60 (9.26) | -2.78 (-5.95, 0.39) | 25.93 (6.84) | 20.92 (9.12) | -5.01 (-7.84, -2.18) | 2.23 (-1.00, 5.47) | 0.18 |
| 35-49y | 22.94 (6.16) | 18.41 (7.66) | -4.52 (-7.27, -1.77) | 24.02 (8.15) | 21.79 (7.62) | -2.23 (-4.84, 0.37) | -2.29 (-5.18, 0.60) | 0.12 |
| 50-78y | 22.66 (7.45) | 20.34 (7.67) | -2.32 (-5.45, 0.80) | 22.93 (8.45) | 20.53 (7.75) | -2.40 (-5.74, 0.94) | 0.08 (-3.41, 3.56) | 0.97 |
| **Marital Status at Baseline** |  |  |  |  |  |  |  |  |
| Never married | 23.67 (6.73) | 20.93 (8.55) | -2.74 (-5.24, -0.24) | 26.36 (8.58) | 21.64 (8.61) | -4.72 (-7.13, -2.31) | 1.98 (-0.66, 4.63) | 0.14 |
| Married | 22.94 (5.92) | 21.08 (6.59) | -1.86 (-5.32, 1.59) | 24.30 (6.14) | 21.74 (7.39) | -2.56 (-5.34, 0.22) | 0.69 (-2.69, 4.07) | 0.69 |
| Divorced, separated, or  widowed | 22.95 (7.03) | 17.36 (8.56) | -5.58 (-8.81, -2.36) | 19.52 (6.79) | 18.86 (8.28) | -0.66 (-4.56, 3.23) | -4.92 (-8.77, -1.07) | 0.012 |
| **Study Park Use at Follow-Up** |  |  |  |  |  |  |  |  |
| High (≥ Once per week) | 23.18 (6.76) | 19.06 (7.17) | -4.12 (-6.34, -1.90) | 22.85 (6.94) | 20.04 (7.69) | -2.81 (-5.10, -0.52) | -1.31 (-3.73, 1.12) | 0.29 |
| Low (< Once per week) | 23.44 (6.38) | 21.36 (9.56) | -2.09 (-4.87, 0.70) | 26.17 (8.45) | 22.47 (8.54) | -3.71 (-6.14, -1.27) | 1.62 (-1.20, 4.44) | 0.26 |
| ^1^Mean (SD)  ^2^Estimated using linear mixed effects regression models adjusted for education, public housing, and marital status at baseline  ^3^The DID estimator represents the difference in change in mean PSS score in the intervention group minus the control group  Abbreviations – PSS: Perceived Stress Scale; PARCS: Physical Activity and Redesigned Community Spaces; DID: Difference-in-Difference | | | | | | | | |

**Table 4. Changes in perceived stress over time among adult PARCS study participants in intervention vs. control groups after additionally adjusting for study park use**

|  | **Intervention**  **(n = 162)** | | | **Control**  **(n = 151)** | | | **Difference-in-Differences** | |
| --- | --- | --- | --- | --- | --- | --- | --- | --- |
|  | **Pre-Renovation**^1^ | **Post-Renovation**^1^ | **Change**  **(95% CI)** ^2^ | **Pre-Renovation**^1^ | **Post-Renovation**^1^ | **Change**  **(95% CI)** ^2^ | **DID Estimator (95% CI)^2,3^** | **p-value** |
| **Overall Sample** | 23.25 (6.85) | 20.37 (8.23) | -2.88 (-4.48, -1.29) | 24.45 (7.81) | 21.28 (8.08) | -3.16 (-4.82, -1.51) | 0.28 (-1.48, 2.03) | 0.75 |
| **Age at Baseline** |  |  |  |  |  |  |  |  |
| 18-34y | 24.39 (6.84) | 22.08 (8.91) | -2.32 (-5.08, 0.45) | 25.93 (6.84) | 20.92 (9.12) | -5.01 (-7.89, -2.14) | 2.70 (-0.34, 5.73) | 0.082 |
| 35-49y | 23.06 (6.34) | 19.39 (7.66) | -3.67 (-6.21, -1.13) | 24.13 (8.12) | 21.87 (7.46) | -2.26 (-4.80, 0.28) | -1.41 (-4.14, 1.33) | 0.31 |
| 50-78y | 22.15 (7.49) | 19.70 (8.04) | -2.45 (-5.51, 0.62) | 22.99 (8.34) | 20.76 (7.77) | -2.23 (-5.57, 1.11) | -0.22 (-3.67, 3.23) | 0.90 |
| **Marital Status at Baseline** |  |  |  |  |  |  |  |  |
| Never married | 23.76 (7.16) | 21.37 (8.21) | -2.39 (-4.64, -0.14) | 26.36 (8.58) | 21.64 (8.61) | -4.72 (-7.16, -2.28) | 2.33 (-0.20, 4.85) | 0.071 |
| Married | 22.75 (5.99) | 21.57 (6.67) | -1.19 (-4.37, 1.99) | 24.08 (6.11) | 21.83 (7.27) | -2.25 (-4.98, 0.49) | 1.06 (-2.13, 4.26) | 0.51 |
| Divorced, separated or  widowed | 22.77 (7.09) | 17.33 (9.01) | -5.44 (-8.54, -2.34) | 20.64 (7.46) | 19.43 (8.24) | -1.22 (-4.95, 2.52) | -4.22 (-7.92, -0.53) | 0.025 |
| ^1^Mean (SD)  ^2^Estimated using linear mixed effects regression models adjusted for education, public housing, and marital status at baseline, and study park use at follow-up  ^3^The DID estimator represents the difference in change in mean PSS score in the intervention group minus the control group  Abbreviations – PSS: Perceived Stress Scale; PARCS: Physical Activity and Redesigned Community Spaces; DID: Difference-in-Difference | | | | | | | | |

**Table 5. Changes in perceived stress over time among adult PARCS study participants by intervention status and study park use at follow-up, with high study park use defined as “> Once per week” and low study park use defined as “≤ Once per week”**

| **Overall Sample** | | | | | |
| --- | --- | --- | --- | --- | --- |
|  |  | **Intervention** | **Control** |  | **Difference** |
|  |  | **(n = 162)^1^** | **(n = 151)^1^** |  | **(Intervention - Control)^2^** |
| **Study Park Use at Follow-Up** |  |  |  |  |  |
| High (> Once per week) (n = 133) |  | -3.49 (-5.84, -1.14) | -2.21 (-4.88, -0.46) |  | -1.28 (-3.99, 1.43) |
| Low (≤ Once per week) (n = 180) |  | -2.36 (-4.54, -0.18) | -3.76 (-5.87, -1.65) |  | 1.39 (-0.92, 3.71) |
|  |  |  |  |  |  |
| **Difference (High - Low)^3^** |  | -1.13 (-3.57, 1.31) | 1.54 (-1.05, 4.14) |  | 3-way interaction^4^  p = 0.14 |
|  |  |  |  |  |  |
| **Age 18-34y** | | | | | |
|  |  | **Intervention** | **Control** |  | **Difference** |
|  |  | **(n = 54)^1^** | **(n = 50)^1^** |  | **(Intervention - Control)^2^** |
| **Study Park Use at Follow-Up** |  |  |  |  |  |
| High (> Once per week) (n = 35) |  | -2.99 (-6.65, 0.67) | -5.73 (-9.49, -1.97) |  | 2.74 (-2.51, 7.99) |
| Low (≤ Once per week) (n = 69) |  | -1.98 (-4.56, 0.61) | -4.64 (-7.34, -1.94) |  | 2.66 (-1.08, 6.40) |
|  |  |  |  |  |  |
| **Difference (High - Low)^3^** |  | -1.01 (-5.49, 3.47) | -1.09 (-5.72, 3.55) |  | 3-way interaction^4^  p = 0.98 |
|  |  |  |  |  |  |
| **Age 35-49y** | | | | | |
|  |  | **Intervention** | **Control** |  | **Difference** |
|  |  | **(n = 64)^1^** | **(n = 64)^1^** |  | **(Intervention - Control)^2^** |
| **Study Park Use at Follow-Up** |  |  |  |  |  |
| High (> Once per week) (n = 55) |  | -4.94 (-7.88, -2.01) | -0.56 (-3.55, 2.42) |  | -4.38 (-8.57, -0.19) |
| Low (≤ Once per week) (n = 73) |  | -2.67 (-5.26, -0.09) | -3.50 (-6.05, -0.95) |  | 0.83 (-2.81, 4.46) |
|  |  |  |  |  |  |
| **Difference (High - Low)^3^** |  | -2.27 (-6.18, 1.64) | 2.93 (-0.99, 6.86) |  | 3-way interaction^4^  p = 0.066 |
|  |  |  |  |  |  |
| **Age 50-78y** | | | | | |
|  |  | **Intervention** | **Control** |  | **Difference** |
|  |  | **(n = 44)^1^** | **(n = 37)^1^** |  | **(Intervention - Control)^2^** |
| **Study Park Use at Follow-Up** |  |  |  |  |  |
| High (> Once per week) (n = 43) |  | -2.40 (-5.28, 0.48) | -1.12 (-5.27, 3.03) |  | -1.28 (-6.33, 3.77) |
| Low (≤ Once per week) (n = 38) |  | -2.54 (-6.55, 1.47) | -2.90 (-6.14, 0.33) |  | 0.36 (-4.79, 5.51) |
|  |  |  |  |  |  |
| **Difference (High - Low)^3^** |  | 0.14 (-4.80, 5.08) | 1.78 (-3.48, 7.04) |  | 3-way interaction^4^  p = 0.65 |

Estimates in table are from linear mixed effects regression models adjusted for education, public housing, and marital status at baseline

^1^Mean change in PSS (post-renovation mean – pre-renovation mean) and 95% CI

^2^Difference in mean change in PSS between intervention and control groups and 95% CI

^3^Difference in mean change in PSS between high and low park use groups and 95% CI

^4^p-value is for 3-way interaction between intervention status x time x study park use at follow-up

Abbreviations – PSS: Perceived Stress Scale; PARCS: Physical Activity and Redesigned Community Spaces

**Table 6. Changes in perceived stress over time among adult PARCS study participants by intervention status and study park use at follow-up, with high study park use defined as “> Once per month” and low study park use defined as “≤ Once per month”**

| **Overall Sample** | | | | | |
| --- | --- | --- | --- | --- | --- |
|  |  | **Intervention** | **Control** |  | **Difference** |
|  |  | **(n = 162)^1^** | **(n = 151)^1^** |  | **(Intervention - Control)^2^** |
| **Study Park Use at Follow-Up** |  |  |  |  |  |
| High (> Once per month) (n = 204) |  | -3.80 (-5.78, -1.82) | -3.20 (-5.23, -1.16) |  | -0.60 (-2.77, 1.56) |
| Low (≤ Once per month) (n = 109) |  | -1.20 (-3.89, 1.49) | -3.10 (-5.92, -0.29) |  | 1.90 (-1.06, 4.87) |
|  |  |  |  |  |  |
| **Difference (High - Low)^3^** |  | -2.60 (-5.14, -0.06) | -0.09 (-2.74, 2.55) |  | 3-way interaction^4^  p = 0.18 |
|  |  |  |  |  |  |
| **Age 18-34y** | | | | | |
|  |  | **Intervention** | **Control** |  | **Difference** |
|  |  | **(n = 54)^1^** | **(n = 50)^1^** |  | **(Intervention - Control)^2^** |
| **Study Park Use at Follow-Up** |  |  |  |  |  |
| High (> Once per month) (n = 66) |  | -4.57 (-7.38, -1.75) | -5.27 (-7.84, -2.70) |  | 0.70 (-3.11, 4.51) |
| Low (≤ Once per month) (n = 38) |  | 0.50 (-2.65, 3.65) | -4.36 (-8.48, -0.24) |  | 4.86 (-0.33, 10.04) |
|  |  |  |  |  |  |
| **Difference (High - Low)^3^** |  | -5.06 (-9.29, -0.84) | -0.91 (-5.77, 3.95) |  | 3-way interaction^4^  p = 0.20 |
|  |  |  |  |  |  |
| **Age 35-49y** | | | | | |
|  |  | **Intervention** | **Control** |  | **Difference** |
|  |  | **(n = 64)^1^** | **(n = 64)^1^** |  | **(Intervention - Control)^2^** |
| **Study Park Use at Follow-Up** |  |  |  |  |  |
| High (> Once per month) (n = 76) |  | -4.75 (-7.25, -2.25) | -1.64 (-4.14, 0.86) |  | -3.10 (-6.64, 0.43) |
| Low (≤ Once per month) (n = 52) |  | -2.09 (-5.11, 0.94) | -3.16 (-6.19, -0.14) |  | 1.08 (-3.20, 5.35) |
|  |  |  |  |  |  |
| **Difference (High - Low)^3^** |  | -2.66 (-6.59, 1.26) | 1.52 (-2.41, 5.44) |  | 3-way interaction^4^  p = 0.14 |
|  |  |  |  |  |  |
| **Age 50-78y** | | | | | |
|  |  | **Intervention** | **Control** |  | **Difference** |
|  |  | **(n = 44)^1^** | **(n = 37)^1^** |  | **(Intervention - Control)^2^** |
| **Study Park Use at Follow-Up** |  |  |  |  |  |
| High (> Once per month) (n = 62) |  | -2.20 (-4.74, 0.33) | -2.57 (-5.66, 0.51) |  | 0.37 (-3.62, 4.36) |
| Low (≤ Once per month) (n = 19) |  | -3.73 (-9.55, 2.10) | -1.51 (-5.96, 2.94) |  | -2.22 (-9.55, 5.12) |
|  |  |  |  |  |  |
| **Difference (High - Low)^3^** |  | 1.52 (-4.83, 7.88) | -1.06 (-6.48, 4.35) |  | 3-way interaction^4^  p = 0.54 |

Estimates in table are from linear mixed effects regression models adjusted for education, public housing, and marital status at baseline

^1^Mean change in PSS (post-renovation mean – pre-renovation mean) and 95% CI

^2^Difference in mean change in PSS between intervention and control groups and 95% CI

^3^Difference in mean change in PSS between high and low park use groups and 95% CI

^4^p-value is for 3-way interaction between intervention status x time x study park use at follow-up

Abbreviations – PSS: Perceived Stress Scale; PARCS: Physical Activity and Redesigned Community Spaces
